# Positron Emission Tomography in Takayasu Arteritis: A Review Including Patterns of Vascular Involvement Across Modalities and Regions

**DOI:** 10.3390/jcm14092939

**Published:** 2025-04-24

**Authors:** Tokio Katakura, Tsuyoshi Shirai

**Affiliations:** Department of Rheumatology, Tohoku University Hospital, 1-1 Seiryo-machi, Aoba-ku, Sendai 980-8574, Miyagi, Japan

**Keywords:** computed tomography, imaging, positron emission tomography, Takayasu arteritis, vasculitis

## Abstract

Takayasu arteritis (TAK) is a rare, chronic large-vessel vasculitis that predominantly affects the aorta and its major branches. Early and accurate diagnosis remains essential to prevent irreversible vascular damage and organ dysfunction. Positron emission tomography/computed tomography (PET/CT) has emerged as a valuable imaging modality for detecting active vascular inflammation in TAK. Using 18F-fluorodeoxyglucose (18F-FDG), PET/CT enables the assessment of metabolic activity in inflamed arterial walls, supporting both initial diagnosis and disease monitoring. Compared with conventional imaging techniques, such as magnetic resonance imaging (MRI) and computed tomography (CT), PET/CT provides functional data correlated with inflammatory activity rather than solely anatomical changes. Recent studies have highlighted its utility in distinguishing active from chronic disease, predicting relapse, and evaluating treatment response. This review summarizes the role of PET/CT in TAK, addressing its advantages, patterns of vascular involvement, limitations, and future perspectives. Vascular lesions identified using PET/CT do not always align with those detected by other imaging modalities, with PET/CT demonstrating superiority in revealing aortic inflammation potentially overlooked by alternative techniques. Further research is needed to establish whether PET/CT-based vascular involvement patterns, rather than conventional angiographic findings, can help identify disease subtypes of TAK.

## 1. Introduction

Takayasu arteritis (TAK) is a type of large vessel vasculitis (LVV) predominantly affecting the aorta and its major branches in young patients [1]. Although the precise pathomechanism of TAK remains under investigation, multiple inflammatory cells—including macrophages, T cells, B cells, and NK cells—are activated and contribute to its pathogenicity [2,3]. Chronic inflammation in TAK damages the aorta and its major branches, resulting in dilatation and stenosis of the affected arteries, occasionally necessitating surgical intervention [4]. Traditionally, vascular assessment of TAK has relied on modalities such as catheter angiography, ultrasonography, computed tomography (CT), and magnetic resonance imaging (MRI) [5]. In recent years, however, positron emission tomography/computed tomography (PET/CT) has gained recognition for its effectiveness in evaluating TAK. Imaging with [¹⁸F] fluorodeoxyglucose (FDG)-PET relies on the elevated glucose demand of inflammatory cells in vascular inflammation [6]. Initially, it was applied to vascular inflammation to identify high-risk lesions and quantify the disease burden of atherosclerosis [7]. Subsequently, PET/CT demonstrated potential utility in the assessment of LVV. Compared to other modalities, PET/CT offers the advantage of directly evaluating vascular inflammation, which may aid in predicting future vascular changes. Despite this, no consensus exists regarding its diagnostic accuracy, assessment of disease activity, pathophysiological evaluation, or prognostic value. This review comprehensively examines research on PET/CT in TAK, discusses current findings, and explores its future prospects. Moreover, differences in affected arteries across modalities and regions are evaluated.

## 2. FDG-PET/CT as a Diagnostic Tool

The diagnostic performance of FDG-PET/CT for TAK has been extensively studied, with numerous reviews and meta-analyses available on this topic. The foundation for this research stems from a case report published in 1999, which documented FDG uptake in a patient with TAK corresponding to areas of wall thickening in the aorta and its branches. This uptake was observed at an early stage, prior to significant stenosis or aortic aneurysm development [8]. The potential utility of PET/CT for the early diagnosis of TAK has been suggested, even in the absence of luminal changes. Since then, numerous clinical studies have been conducted; however, most share common limitations, including small study populations, retrospective observational designs, and control groups comprising either healthy individuals or patients with cancer. Notably, FDG uptake in the axillary and femoral arteries is frequently observed when patients with atherosclerosis are selected as controls [9]. Among studies evaluating diagnostic performance, the largest reported meta-analysis included eight studies encompassing 170 patients with LVV and 230 controls. This analysis reported a diagnostic sensitivity of 75.9% (95% confidence interval [CI] 68.7–82.1), with specificity at 93.0% (95% CI 88.9–96.0). The area under the curve (AUC) was 0.863 and the Q* index was 0.794, indicating good diagnostic accuracy. However, caution is warranted, as this analysis treated LVV as a composite entity with giant cell arteritis (GCA), with only 19 of the 170 patients diagnosed with TAK [10]. When restricted to three studies involving patients with GCA alone, sensitivity and specificity were reported as 83.3% (95% CI: 72.1–91.4) and 89.6% (95% CI: 79.7–95.7), respectively. Additionally, another meta-analysis of four studies, limited to GCA alone (including 57 GCA patients and 176 controls), reported a diagnostic sensitivity of 89.5% (95% CI: 78.5–96.0) and a specificity of 97.7% (95% CI: 94–99) [11]. While these findings may suggest a reduced diagnostic performance of PET/CT for TAK alone, it is important to note that in GCA as well, diagnostic accuracy tends to decrease when the condition is limited to the cranial region—referred to as cranial GCA—making direct comparisons less appropriate.

Regarding the diagnostic performance of other modalities for TAK, a separate meta-analysis reported sensitivity and specificity values of 81% and 100% for ultrasound, 95% and 100% for CT angiography (CTA), and 79% and 97% for MRI angiography (MRA), respectively. However, control groups varied across modalities [12]. Although PET/CT may not surpass other modalities in overall diagnostic accuracy, particularly in the inactive stage, its ability to detect early-stage TAK before vascular changes develop remains an advantage.

Furthermore, differentiating aortitis from atheroma using PET/CT is often challenging, particularly in older patients with GCA. Studies suggest that FDG uptake intensity and extent play a crucial role in distinguishing between these conditions [13]. Similarly, differentiation from atherosclerosis is important in TAK, especially in older patients. Moreover, because vascular Behçet’s disease, relapsing polychondritis, and G-CSF-associated aortitis can also cause large-vessel inflammation [14], diagnosis should be based not only on imaging findings but also on comprehensive clinical evaluation. In addition, the influence of glucocorticoid (GC) therapy on diagnostic accuracy warrants consideration. In a prospective study involving 24 patients with GCA, all participants received 60 mg of prednisolone daily and underwent PET/CT scans 3 or 10 days after treatment initiation. While GC therapy reduced FDG uptake in large vessels, large-vessel GCA was accurately diagnosed in all patients on Day 3, but only in 5 of 14 patients on Day 10 (*p* < 0.001) [15]. Another retrospective cohort study of 85 patients with GCA, in which the principal reasons for initiating GC treatment before PET/CT were visual manifestations or cerebrovascular events at diagnosis (43.5%), reported a significant negative correlation between PET scores and both the duration and dose of GC therapy. Nonetheless, PET/CT performed after 10 days of treatment remained diagnostically useful in a substantial number of cases [16]. Ideally, PET/CT should be performed before or soon after treatment initiation based on clinical urgency.

Despite the limited number of studies on PET/CT imaging in anti-neutrophil cytoplasmic antibody (ANCA)-associated vasculitis (AAV), and the even scarcer data on polyarteritis nodosa [17], most findings suggest its potential diagnostic value. In a retrospective study of granulomatosis with polyangiitis (GPA), 44 PET scans performed in 33 patients during suspected active disease revealed PET-positive lesions, most frequently in the nasopharynx (*n* = 22) and lungs (*n* = 22), with no uptake observed in small- or medium-sized vessels themselves. Interestingly, PET/CT also identified 41 clinically occult lesions, including aortic involvement in eight cases [18]. Involvement of large vessels in AAV, although relatively uncommon, constitutes a clinically important presentation as it can influence treatment decisions and affect clinical outcomes [19,20]. Nevertheless, caution is warranted as PET/CT may not detect all disease manifestations. In a retrospective study of 14 patients with AAV (eight with GPA), PET identified lesions in sinonasal, lung, cardio-vascular, and kidney tissues, whereas skin, joint, eye and peripheral nervous system impairments remained undetected [21]. Several studies have reported a reduction in FDG uptake following treatment in AAV, supporting the potential role of PET in identifying lesions suitable for biopsy or requiring close monitoring during follow-up [18,22]. In clinical practice, it is often challenging to determine whether newly emerging abnormal imaging findings during the treatment of GPA—particularly in the lungs—are due to disease activity, malignancy, or infection. In a retrospective cohort study of 26 patients with AAV (15 with GPA), PET/CT was performed to differentiate these possibilities. Notably, FDG uptake was more frequently observed in cases of infection or malignancy than in cases of disease relapse, with a negative predictive value of 93% (95% CI: 68–99%) [23].

## 3. Activity Assessment

Disease activity assessment using PET/CT has been evaluated in a meta-analysis of seven studies, encompassing 191 patients with TAK (96 with active disease). PET/CT distinguished active disease from clinical remission with a sensitivity of 87% (95% CI, 78.0–92.6) and a specificity of 73% (95% CI, 62.5–81.3) [11]. Another study reported sensitivity and specificity values of 81% (95% CI: 69–89%) and 74% (95% CI: 55–86%), respectively [12]. Clinical disease activity in these studies was primarily assessed using NIH criteria [24]. Persistent FDG uptake has been noted in some patients during remission, potentially contributing to the lower specificity of PET/CT for disease activity. This observation raises an unresolved issue: whether PET/CT-defined activity aligns with clinical disease activity [25].

The definition of PET/CT-based disease activity varies between studies, though two primary approaches predominate: one based on the maximal standardized uptake value (SUVmax) of affected vessels and another based on the summation of FDG uptake across selected vascular lesions. Studies focusing on the SUVmax have reported similar findings. A single-center retrospective study from Japan, involving 39 patients with TAK (27 with active disease), established an SUVmax cutoff of 2.1, achieving a sensitivity of 92.6% and a specificity of 91.7% [26]. Similarly, a single-center retrospective study from China, also involving 39 patients with TAK (29 with active disease), determined that an SUVmax cutoff of 2.2 differentiated active disease with a sensitivity of 86% and a specificity of 90% [27]. A simpler approach has been reported in which FDG uptake equal to or greater than that of the liver is used as a criterion for PET/CT-based disease activity [28]. Studies focusing on the sum of the FDG uptake across selected vascular lesions have reported similar findings. In 2006, a prospective study involving 35 patients with GCA introduced a method called the total vascular score. Large vessels and their branches were divided into seven segments, and FDG uptake was semi-quantitatively scored on a four-point scale, with the total score calculated [29]. Although this method has not been validated for TAK, it remains a promising approach. In 2018, another group proposed a new method based on a prospective longitudinal cohort of patients with LVV, including TAK, employing a relevant comparator group as a control [9]. The disease activity assessment index reported by Grayson et al., known as the PETVAS, involves dividing the aorta and its branches into 15 segments and assessing FDG uptake using a four-point semi-quantitative scale. A PETVAS threshold of ≥20 differentiated disease activity with a sensitivity of 68% (95% CI: 50–83%) and a specificity of 71% (95% CI: 58–82%). A recent retrospective observational study from India, involving 36 patients with TAK, found that an SUVmax cutoff of 2.2 differentiated disease activity with a sensitivity of 68% and a specificity of 100% (AUC = 0.84), while a PETVAS cutoff of 5.5 demonstrated comparable diagnostic accuracy, with a sensitivity of 50% and a specificity of 100% (AUC = 0.75) [30]. Notably, the PETVAS cutoff in this study differed significantly from the original threshold, with the authors suggesting that Asian patients with TAK may exhibit lower PETVAS values. An unresolved pathophysiological question persists: whether PETVAS correlation with disease activity primarily reflects severe focal inflammation (i.e., high SUVmax) at specific sites or the extent of vascular involvement, warranting further investigation.

## 4. Monitoring Activity

PET/CT is employed to monitor disease activity; however, persistent FDG uptake is frequently observed, even in clinically inactive or remissive cases, complicating result interpretation. A prospective cohort study tracking clinical symptoms, laboratory findings, and imaging changes, analyzed 112 patients with LVV (including 56 patients with TAK). In patients transitioning from active disease to remission, the erythrocyte sedimentation rate, C-reactive protein level, and PETVAS score decreased concurrently. However, regarding PETVAS, FDG uptake did not completely resolve (active phase: 23 (interquartile range (IQR), 16.5–25) vs. remission phase: 17.5 (IQR, 12–21.3), *p* < 0.01) [31]. A single-center retrospective study examining longitudinal PETVAS changes reported a significant decline over time in GCA, whereas no such reduction was observed in TAK. Given that many included patients with TAK had prolonged disease duration, early-phase changes might not have been detected [32]. The relationship between FDG uptake in the absence of clinical disease activity and clinical outcomes remains a critical area for future research. In clinical practice, monitoring vascular injury with complementary modalities aids in preventing disease progression. An increase in FDG uptake compared with a previous scan suggests active inflammation, necessitating appropriate follow-up or intervention.

Relapse occurs frequently in TAK, affecting 60% of cases [33]. Recent use of biologics facilitates GC dose reduction [34] and offers potential for GC-free remission in certain patients [35]. A challenge in managing biologics, such as tocilizumab (TCZ), which inhibits the interleukin 6 pathway, lies in their suppression of C-reactive proteins, hindering disease activity monitoring. Isobe et al. evaluated PET/CT efficacy in detecting inflammation recurrence during TCZ treatment, finding that PET/CT findings closely aligned with the clinical course in all relapsed patients, even during TCZ therapy, while scans remained negative in not-relapsed patients [36]. Therefore, PET/CT appears suitable for detecting disease activity in patients receiving TCZ. A major concern when using PET/CT for treatment response assessment in TAK is the use of prosthetic materials, such as artificial valves and grafts, which are implanted because of surgical interventions. These prosthetic materials often exhibit FDG uptake, complicating vasculitis evaluation. In a prospective observational study involving 26 patients with TAK after graft surgery, periprosthetic FDG uptake was significant in 23 patients, with a mean SUVmax of 4.2 ± 1.5. The median periprosthetic FDG uptake semiquantitative score was 3 (IQR, 3–3); however, MRA did not reveal arterial progression in 25 patients [37]. Similarly, a retrospective observational study involving 30 TAK patients reported that significant periprosthetic FDG uptake was observed in six of seven patients (86%) with previous vascular surgery and in 10 of 11 grafts (91%) [38].

## 5. Prediction for Prognosis

The ability of PET/CT to predict the prognosis of vascular injury remains a topic of great interest, with inconsistent findings across studies. The original PETVAS study demonstrated its utility not only in assessing disease activity but also in predicting subsequent relapse [9]. Patients with a PETVAS ≥20 exhibited a higher likelihood of relapse. However, most relapses (91%) occurred in patients with GCA, and the study population—particularly those with TAK—had a prolonged disease duration (median 12.5 years) and prior pharmacological interventions. A single-center retrospective observational study from Italy, involving 100 patients with LVV (49 with TAK), found that a PETVAS ≥10 differentiated clinically active from inactive cases with a sensitivity of 60.8% and a specificity of 80.6%. However, no association with subsequent relapse was observed (hazard ratio (HR) = 1.04; 95% CI 1.0–1.1). In contrast to the original PETVAS study, 56% (19 cases) of relapses occurred in patients with TAK, with 84% (16 cases) detected through radiographic progression [39]. The discrepancy may stem from the fact that, in both studies, most patients with TAK had received prior treatment, potentially modifying clinical disease activity at the time of PET/CT assessment due to disease duration or therapeutic interventions. A prospective cohort study from China, focusing on newly diagnosed patients with TAK, examined a more homogeneous population. A baseline PETVAS ≥8 was identified as an independent risk factor for new angiographic vascular lesions (HR = 7.56, 95% CI 2.2–26.01; *p* < 0.01) [40]. As discussed later, the distribution of affected vessels and the clinical presentation of TAK may vary by racial background, highlighting the need for further validation through similar investigations. In addition to PETVAS, other predictors of relapse have also been explored. A single-center retrospective study from Brazil determined that patients with a baseline SUVmax ≥ 1.3 faced a significantly higher risk of disease relapse compared to those with a lower SUVmax (85.0% vs. 50.0%, *p* = 0.049, odds ratio 5.667, 95% CI: 1.07–30.09) [41]. In a prospective cohort study involving 70 patients with LVV (including 38 with TAK), new vascular lesions developed in 13% of patients with TAK, while progression of existing lesions was observed in 8%. Notably, this study found that in arterial territories that developed angiographic changes, the majority exhibited PET/CT activity at baseline. Although no significant difference in SUVmax was found between paired arterial territories with and without lesion progression, those with progression displayed a higher frequency of wall thickening and edema at baseline [42]. A similar finding is addressed later [43]. Intriguingly, vascular changes occurred even in arterial territories without high baseline PET scores at baseline, a result that warrants particular attention.

## 6. Frequency and Distribution of Affected Vessels in FDG-PET

### 6.1. Frequency

**Figure 1:**
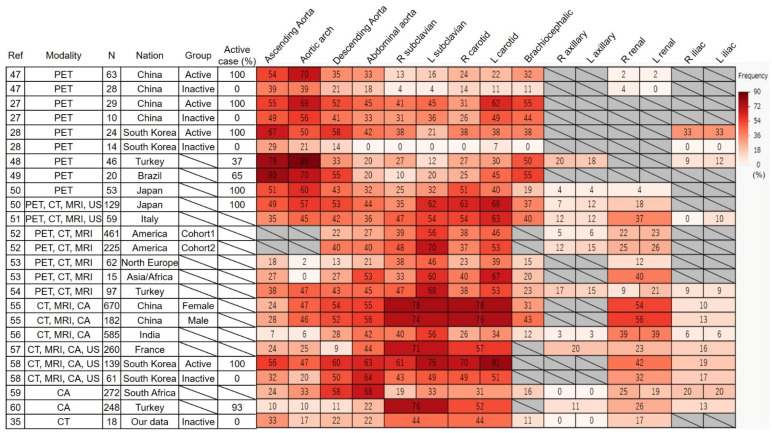
The frequency of arterial territory in Takayasu arteritis detected by several modalities in the literature is summarized as a heat map. Values are presented as percentages (%), with darker red indicating higher values. Abbreviations: CA, conventional angiography; CT, computed tomography; MRI, magnetic resonance imaging; PET, positron emission tomography; US, ultrasound.

The incidence of TAK and the prevalence of HLA-B52 positivity vary across countries [44]. Patients with TAK occasionally present with coexisting inflammatory conditions such as ulcerative colitis (relatively common) [2], or osteomyelitis [45] and glomerulonephritis (rare), underscoring the disease’s heterogeneity. Conversely, identical gut microbiota abnormalities have been identified across diverse environments [46], suggesting a shared basis for disease onset. As a form of LVV, TAK induces inflammation in the aorta and its major branches, with the frequency of affected blood vessels differing by region. Therefore, we conducted a systematic literature review on the frequency of vascular involvement and its association with imaging modalities. A comprehensive search of PubMed and Google Scholar identified studies investigating TAK and reporting the frequency of vascular involvement. Five studies utilized PET/CT alone, five combined PET/CT with additional imaging modalities, and 24 examined non-PET modalities, among which, those related to PET/CT or that had a large sample size were included in our analysis (Figure 1) [27,28,35,47,48,49,50,51,52,53,54,55,56,57,58,59,60].

The frequency of affected arterial lesions detected using PET/CT followed this pattern: ascending aorta and aortic arch > brachiocephalic artery, carotid artery, and descending aorta > abdominal aorta > subclavian artery. Furthermore, FDG uptake was observed less frequently in inactive patients than in active patients. Although the frequency varied slightly across studies, no marked differences were observed, whereas differences across imaging modalities were clearly identified. PET/CT alone preferentially detected lesions in the ascending aorta and aortic arch (approximately 60%), compared with a lower detection rate (30%) in studies using composite modalities. In contrast, subclavian and carotid artery lesions were less frequently identified using PET/CT alone (25%), but more commonly detected with other modalities (55%). The spatial resolution of PET/CT suggests that FDG uptake is influenced by confounding factors such as vessel diameter and wall thickness [61]. The intensity of FDG uptake correlates with the number of inflammatory cells in the lesion, potentially leading to underestimation in smaller arteries by PET/CT. Moreover, differentiating slight uptake due to atherosclerotic changes remains challenging, as noted earlier.

In addition, the discrepancies in affected lesions between modalities may be reflected in the methods used to detect the lesions. Widely recognized angiographic definitions of vascular lesions—such as stenosis, occlusion, and aneurysm [62]—do not represent the earliest changes in large arteries. In particular, stenosis and occlusion are more frequently observed in smaller arteries. However, aortic aneurysms and regurgitation associated with the ascending aortic dilatation are major complications that significantly affect the prognosis of TAK [63]. Differences in imaging modalities’ ability to detect vascular lesions have been explored in previous studies. Grayson et al. compared the utility of PET and MRI in patients with LVV, finding high concordance between the two, particularly in detecting vascular edema and wall thickening. However, MRI demonstrated superior sensitivity for luminal abnormalities such as stenosis, suggesting it may be more effective in evaluating structural vascular involvement. In contrast, PET appears to correlate more strongly with clinical disease activity, indicating its potential utility for activity assessment [64]. These findings suggest that incorporating PET/CT observations of the ascending aorta and aortic arch—even without luminal abnormalities like aneurysms or stenosis—into the assessment of disease activity warrants further exploration. Moreover, early wall thickening, which is commonly observed in the aorta, is often overlooked in current classification and activity assessment criteria, representing another critical issue that should be addressed in future studies. The Japanese diagnostic criteria for TAK [65] incorporated arterial wall thickness as the content of arterial inflammation. In a retrospective observational study of 100 patients with LVV (including 53 with TAK), morphological wall thickening identified using CT or MRA did not predict subsequent stenosis or dilatation. However, a strong correlation was observed between wall thickening and FDG uptake, with PET/CT scores predicting future vascular complications, such as stenosis or dilatation, during the follow-up. This may indicate that PET/CT distinguishes metabolically active inflammatory wall thickening from chronic fibrotic wall thickening without inflammatory activity, with the former being more likely to precede vascular damage [43].

### 6.2. Distribution

The distribution of affected vessels was analyzed using 130 PET/CT scans from 28 patients with LVV (including 13 with TAK). Three distinct clusters of vascular involvement in TAK were identified: (1) femoral and iliac arteries; (2) aortic arch, ascending, descending, and abdominal aorta; and (3) carotid, subclavian, and axillary arteries [43]. Furthermore, in another study analyzing the distribution of affected vessels using multiple imaging modalities, including PET, 1068 patients with LVV (including 686 with TAK) were evaluated. This study identified three distinct vascular involvement patterns in TAK compared to GCA: (1) abdominal vasculature involvement, (2) bilateral disease of the subclavian and carotid arteries, and (3) focal disease confined to the left subclavian artery. In contrast, GCA exhibited more diffuse involvement, such as bilateral axillary/subclavian arteries, or minimal disease without a clear pattern [52]. Although vascular involvement in TAK has long been classified into distinct patterns [66], PET/CT imaging may reveal alternative clusters differing from traditional descriptions. Whether these imaging-based patterns correlate with underlying pathophysiology or clinical outcomes remains uncertain. We previously identified two novel autoantibodies in TAK—an anti-endothelial protein C receptor antibody and an anti-scavenger receptor class B type 1 antibody—and demonstrated that these autoantibodies may be associated with distinct clinical features and patterns of vascular involvement [67]. Further studies are needed to explore the relationship between the vascular distribution based on PET/CT-defined active inflammation, novel autoantibodies, and clinical outcomes.

## 7. Differences in Affected Lesions Between Countries

When multiple imaging modalities—including CT, MRI, and ultrasound, not limited to PET/CT—were considered, the frequency of affected arterial territories in TAK exhibited geographical variations (Figure 1), although the overall trend was carotid and subclavian arteries > thoracoabdominal aorta. Japan, Italy, and Turkey showed high frequencies of carotid (63–68%, 54–63%, and 38–53%, respectively) and subclavian artery involvement (35–62%, 47–54%, and 47–68%, respectively), alongside moderate thoracoabdominal aorta involvement (49–53%, 35–45%, and 38–47%, respectively). Japan notably demonstrates a lower frequency of renal artery involvement (18%) compared with other countries. In contrast, Northern Europe and Asia/Africa showed lower frequencies of thoracic aortic involvement (2–18% and 0–27%, respectively) than carotid (23–39% and 40–67%, respectively) and subclavian arteries (38–46% and 33–60%, respectively). Asia/Africa, however, exhibited higher frequencies of abdominal aortic involvement (53%) and renal artery involvement (40%).

Even in studies using composite modalities, excluding PET, the overall pattern of affected arterial lesions showed greater involvement of the carotid and subclavian arteries compared to the thoracoabdominal aorta, although country-specific differences were observed. China and South Korea demonstrated a higher overall frequency of vascular involvement than other countries, potentially due to variations in vascular lesion definitions. India showed a low frequency of ascending aorta (7%) and aortic arch involvement (6%), but a high frequency of renal artery involvement (39%). France was characterized by a low frequency of descending aorta involvement (9%), whereas Africa exhibited a distinct pattern, with higher frequencies of descending (58%) and abdominal aorta involvement (68%) than branch vessels. These data should be interpreted with caution due to population heterogeneity and incomplete imaging evaluations in some cases. Nonetheless, the current classification criteria focus on the clinical manifestations resulting from the involvement of the cervical and upper limb branches, which may not be adequate for identifying patients in some countries or those with specific disease subtypes.

## 8. New Technology

### 8.1. PET/MRI

FDG-PET has recently been integrated with MRI (PET/MRI), with its application in LVV documented [68]. Einspieler et al. reported that SUVmax and visual scores remained consistent between PET/MRI and PET/CT in 12 patients with LVV [69]. As MRI can be used to evaluate the nature of the vascular wall, Laurent et al. proposed three PET/MRI patterns: inflammatory, fibrous, and normal. Combining MRI with FDG-PET offers advantages, including no radiation exposure, improved analysis of vascular arterial gadolinium uptake and simultaneous cardiac evaluation [68].

### 8.2. Somatostatin Receptor 2

As previously noted, FDG-PET exhibits a high false-positive rate in patients with LVV in clinical remission. Ćorović et al. investigated the utility of PET/MRI targeting somatostatin receptor 2 (SST2), which is expressed by inflammatory macrophages, using 68Ga-DOTATATE and 18F-FET-βAG-TOCA [70]. SST2 PET/MRI was generally consistent with FDG-PET/CT imaging in patients with LVV but had a very low background signal in the brain and the heart, allowing for unimpeded assessment of nearby coronary, myocardial, and intracranial artery involvement.

### 8.3. Fibroblast

Röhrich et al. evaluated PET/CT with a novel tracer, 68Ga fibroblast-activated protein inhibitor (FAPI)-46, which monitors nonmalignant conditions, including wound healing, sites of inflammation, atherosclerotic plaques, and diseases leading to fibrosis, as well as malignant conditions [71]. Eight patients with LVV, including three with TAK, were included. FAPI uptake was significantly enhanced in patients with aortitis compared to controls, irrespective of disease activity. Patients in remission exhibited notable FAPI uptake in the vessel walls. Although MRI inflammatory scores were nearly negative in these patients, 80% displayed visually identifiable FAPI uptake, suggesting its potential for monitoring fibrotic disease activity.

## 9. Conclusions

PET/CT proves effective in diagnosing and assessing clinical disease activity in TAK. However, vascular lesions identified using PET/CT do not consistently align with those detected by other imaging modalities. Notably, PET/CT can reveal aortic inflammation overlooked by alternative techniques. While PET/CT reflects inflammatory cell infiltration during the active disease phase, CT and MRI excel at detecting luminal changes—such as stenosis and aneurysms—characteristic of the chronic phase. The accuracy of PET/CT in predicting clinical outcomes remains uncertain, highlighting the need for further studies in treatment-naive populations with minimal vascular changes. A key question for future research is whether PET/CT-based vascular involvement patterns, rather than conventional angiographic findings, can help identify disease subtypes of TAK.

## Data Availability

No new data were created or analyzed in this study.
